# Eating behavior and perception of body shape in Japanese university students

**DOI:** 10.1007/s40519-014-0130-7

**Published:** 2014-05-22

**Authors:** Kumiko Ohara, Yoshiko Kato, Tomoki Mase, Katsuyasu Kouda, Chiemi Miyawaki, Yuki Fujita, Yoshimitsu Okita, Harunobu Nakamura

**Affiliations:** 1 Graduate School of Human Development and Environment, Kobe University, 3-11 Tsurukabuto, Nada, Kobe, Hyogo 657-8501 Japan; 2 Research Fellow of Japan Society for the Promotion of Science, 5-3-1 Kojimachi, Chiyoda, Tokyo, 102-0083 Japan; 3 Department of Childhood Education, Kyoto Seibo College, 1 Fukakusa Taya-cho, Fushimi, Kyoto, 612-0878 Japan; 4 Department of Public Health, Kinki University Faculty of Medicine, 377-2 Oono-Higashi, Osaka-Sayama, Osaka, 589-8511 Japan; 5 Heian Jogakuin (St.Agnes’) College, 5-81-1 Nampeidai, Takatsuki, Osaka, 569-1092 Japan; 6 Graduate School of Science and Technology, Shizuoka University, 3-5-1 Johoku, Naka, Hamamatsu, Shizuoka, 432-8011 Japan

**Keywords:** Body shape, Eating behaviors, Gender differences, Anthropometry measurements

## Abstract

**Purpose:**

We investigated the relationship between eating behavior measured by the Dutch Eating Behaviour Questionnaire (DEBQ) and perception of body shape, examining the current physical status and ‘ideal’ physical parameters in females and males.

**Methods:**

The participants, 548 Japanese university students (age 19.2 ± 0.9 years, mean ± SD; 252 males, 296 females), completed a questionnaire which asked for their current physical status (e.g., weight and height), their ideal physical parameters, their perception of their current body shape, their ideal body shape, and their eating behaviors.

**Results:**

The ideal weight and ideal body mass index (BMI) were significantly higher than the current weight and BMI in the males, but significantly lower in the females. Among the females, the ideal body shape was smaller than their perception of current body shape. The DEBQ scores for restrained, emotional, and external eating were higher in the females than the males among the normal-weight participants, and among the underweight participants, the restrained eating and external eating scores were higher in the females than the males. Restrained eating was negatively associated with the discrepancy between the current and ideal weight, BMI, and body shape in both the males and females. Emotional eating was negatively associated with the discrepancy in current/ideal BMI and body shape only in the females.

**Conclusions:**

At least in Japanese university students, the gender differences in ideal body shape are related to eating behavior.

## Introduction

It is well known that many girls and women around the world feel driven to remain or become thin. Studies of large populations found that the mean body mass index (BMI) of many young females in European countries and the united States is >25 (overweight) [1], and their self-reported ideal body shape was smaller than their current body shape [2]. Young Japanese females have an average BMI of about 20 [3]. However, many of them attempt to achieve a thinner physique as their ideal body shape [4].

In previous studies, eating behavior was found to be associated with body image, and with a drive for thinness. For instance, high score on the Eating Attitude Test-26 (EAT-26) was positively associated with a thin ideal physique or drive for thinness [5, 6]. The EAT-26 was developed by Garner [7] for the screening of patients with eating disorders, in which a score of 20 is considered the clinical cut-off.

A different measure, the Dutch Eating Behaviour Questionnaire (DEBQ), was developed by van Strien to assess eating behavior, including that of normal subjects, in terms of three eating patterns, such as restrained eating, external eating, and emotional eating [8]. However, there are few evidences on the relationship between DEBQ and body shape. Restrained eating of DEBQ was positively associated with the difference between clinical BMI and self-reported BMI [9], positively associated with perception of body weight, which means whether subjects thought they were overweight or underweight [10], and negatively associated with ideal body image [11]. The above reports have referred to only restrained eating of DEBQ. Thus, little is known about an association between DEBQ and body shape from the viewpoint of three eating pattern of DEBQ. In addition, recent studies have also found that many males are preoccupied with their own body shapes [12–14], but there are few studies about males’ perception of body shape, or studies that compare the perception of body shape between males and females. Moreover, in males, the drive for muscularity which is a desire to enhance one’s musculature has been reported [15–18]. Therefore, it is hypothesized that there is a difference in a direction of ideal body shape between males and females or between normal weight and underweight. It is also hypothesized that there is a difference in a relation of DEBQ and body image between males and females or between normal weight and underweight.

In the present study, to address the shortage in the literature, we investigated the relationship between eating behavior as measured by the DEBQ and the perception of body shape between the normal weight and underweight in each gender.

## Methods

### Participants

The survey was conducted using an anonymous, self-administered questionnaire during university classes in 2011–2012. The participants were 618 university students who attended the classes in the liberal arts. Participants were provided no remuneration. The questionnaires were delivered to the all attendance (618 students), and all of them were collected after completion of questionnaire. Out of 618 questionnaires, 548 questionnaires showed valid responses. Thus, the response rate, which was calculated by dividing the valid responses by delivered questionnaires, was 88.7 % (*n* = 548, 252 males and 296 females, 19.2 ± 0.9 years). We classified the participants into three groups according to the World Health Organization (WHO) criteria: overweight (BMI ≥ 25.0 kg/m^2^), normal weight (18.5 ≤ BMI < 25.0 kg/m^2^), and underweight (BMI < 18.5 kg/m^2^). The participants included 94 underweight students (33 males and 61 females), 436 normal-weight students (210 males and 226 females), and 18 overweight students (9 males and 9 females). The overweight individuals were excluded from the present analysis because of small number comparing with the underweight or normal-weight students.

All participants gave informed consent to participate, and the study was approved by the Human Ethics Committee of the Graduate School of Human Development and Environment, Kobe University.

### Measures

The questionnaire identified the participants’ physical status, perception of body shape, and eating behaviors. The physical status questions included four items: age, height, weight, “ideal height,” and “ideal weight” as answered by the participant. Concerning heights and weights, we asked subjects to write down their self-reported heights and weights according to previous reports [19–21]. Each participant’s BMI (kg/m^2^) was calculated by dividing his or her weight in kilograms by the square of the height in meters. Each participant’s “ideal BMI” was calculated in the same way as the BMI using the ideal weight and ideal height he or she provided. A discrepancy of height, weight, and BMI was calculated by “ideal value” minus “current value”.

The participants’ perception of body shape (BSh) was assessed by a 27-item interval scale using four gender-specific BMI-based silhouettes of which validity and reliability have been evaluated in the previous study [22]. The participants were asked to select which single item of the 27-item interval scale most closely represented their actual and ideal body size. A discrepancy of BSh was also calculated by “ideal BSh” minus “current BSh”.

Eating behavior was assessed by the Japanese version of the DEBQ of which validity and reliability have been evaluated in the previous study [23]. The DEBQ is a 33-item self-rated questionnaire and is divided into three subscales: restrained eating (10 items), emotional eating (13 items), and external eating (10 items). Restrained eating means paradoxically dietary restraint (food intake is initially reduced to lose or maintain body weight, but followed by increased consumption and binge eating). Emotional eating means eating in response to negative emotions. External eating means eating in response to the sight or smell of food [24]. The participants were asked to rate each question from 1 for ‘never’ to up to 5 for ‘very often.’ Responses to each question were added together in each subscale, and then divided by the number of questions included in each subscale to produce a score between 1 and 5.

### Statistical analysis

Student’s *t* test was used to evaluate the differences in physical status or DEBQ score between the normal-weight and underweight students in each gender. Pearson’s correlation coefficients were calculated on BMI and each DEBQ scores. A multiple linear regression analysis was used to investigate the association between DEBQ scores and discrepancy of physical index, adjusting for height. A two-way repeated measures analysis of variance (ANOVA) was used to investigate the effects of ‘current’ and ‘ideal’ physical status, the effects of gender, and the interaction effects between ‘current’ and ‘ideal’ physical status and gender on the parameters of physical status. For a post hoc analysis, we used the Bonferroni test. The statistical level for significance was set at 0.05. All statistical analyses were performed by SPSS^®^ 19.0 J for Windows (IBM, Tokyo).

## Results

After excluding the 18 overweight participants, the means and standard deviations of height, weight, and BMI for all participants were 171.6 ± 5.7 cm, 61.1 ± 8.5 kg, and 20.7 ± 2.4 kg/m^2^ in the males, and 157.7 ± 5.5 cm, 50.3 ± 6.5 kg, and 20.2 ± 2.5 kg/m^2^ in the females, respectively. According to the classification of BSh by BMI, 17.7 % (*n* = 94) of the participants were underweight, while 82.3 % (*n* = 436) were normal weight. In the males, 13.1 % (*n* = 33) of the participants were underweight and 83.3 % (*n* = 210) were normal weight. In the females, 20.6 % (*n* = 61) of the participants were underweight and 76.4 % (*n* = 226) were normal weight.

As shown in Table 1, the weight, BMI, perception of BSh, ideal weight, and ideal BMI were all significantly lower in the underweight participants than in the normal-weight participants, in both the males and females (ideal weight in males, *p* = 0.001; other index, *p* < 0.001).

**Table 1:** Physical status of underweight and normal-weight subjects

	Male		Female	
	Underweight (*n* = 33)	Normal weight (*n* = 210)	Underweight (*n* = 61)	Normal weight (*n* = 226)
Height (cm)	171.8 ± 5.7^b^	171.4 ± 5.6^c^	158.5 ± 5.6	157.7 ± 5.4
Weight (kg)	51.9 ± 4.5^ab^	61.6 ± 6.3^c^	44.2 ± 3.7^a^	51.3 ± 4.9
BMI (kg/m^2^)	17.6 ± 0.9^a^	20.9 ± 1.6	17.6 ± 0.9^a^	20.6 ± 1.5
Perception of BSh	7.1 ± 2.4^a^	12.4 ± 3.8	7.5 ± 2.8^a^	12.3 ± 3.4
Ideal height(cm)	176.1 ± 5.2^b^	176.0 ± 5.1^c^	160.1 ± 6.1	159.4 ± 4.5
Ideal weight (kg)	60.0 ± 6.1^ab^	64.9 ± 7.4^c^	44.9 ± 6.5^a^	47.3 ± 4.0
Ideal BMI (kg/m^2^)	19.4 ± 1.5^ab^	20.9 ± 1.8^c^	17.5 ± 1.3^a^	18.6 ± 1.2
Ideal BSh	11.2 ± 3.7^b^	12.1 ± 3.0^c^	6.5 ± 2.8^a^	7.5 ± 2.2
Discrepancy of height (cm)	4.2 ± 4.4^b^	4.6 ± 4.7^c^	1.6 ± 4.7	1.8 ± 3.5
Discrepancy of weight (kg)	8.1 ± 5.7^ab^	3.3 ± 5.9^c^	0.7 ± 7.1^a^	−3.9 ± 3.4
Discrepancy of BMI (kg/m^2^)	1.8 ± 1.4^ab^	−0.03 ± 3.8^c^	−0.1 ± 1.7^a^	−2.0 ± 1.3
Discrepancy of BSh	4.1 ± 3.5^ab^	−0.3 ± 3.8^c^	−1.0 ± 3.9^a^	−4.8 ± 3.1

Discrepancy: ideal value minus current value. Values are means ± standard deviations

*BMI* body mass index, *BSh* body shape

^a^Significantly different from normal weight in each gender (Student’s *t* test with Bonferroni correction)

^b^Significantly different from female underweight (Student’s *t* test with Bonferroni correction)

^c^Significantly different from female normal weight (Student’s *t* test with Bonferroni correction)

Among the females, the perception of ideal BSh was significantly lower in the underweight participants than in the normal-weight participants (*p* = 0.004). In the group of female normal-weight participants, discrepancies of weight, BMI, and BSh were negative, and significantly lower than those in female underweight participants (all three indexes, *p* < 0.001). In male underweight participants, discrepancies of weight, BMI, and BSh were positive, and significantly higher than those in male normal-weight participants (all three indexes, *p* < 0.001). Among all of the underweight participants, the males’ values for height, weight, ideal height, ideal weight, ideal BMI, ideal BSh, discrepancy of height, discrepancy of weight, discrepancy of BMI, and discrepancy of BSh were significantly larger as compared to the females (*p* < 0.05). Similarly, among all of the normal-weight participants, the males’ values for height, weight, ideal height, ideal weight, ideal BMI, ideal BSh, discrepancy of height, discrepancy of weight, discrepancy of BMI, and discrepancy of BSh were significantly larger as compared to the females (*p* < 0.05).

The DEBQ scores for restrained, emotional, and external eating were significantly higher in the females than in the males (all three scores: *p* < 0.001). In addition, as shown in Table 2, the restrained eating values were lower in the underweight participants than in the normal-weight participants in both males and females (males, *p* = 0.003; females, *p* < 0.001). Among all of the underweight participants, the females had significantly higher restrained eating and external eating scores as compared to the males (*p* < 0.05). Among all of the normal-weight participants, the females had significantly higher restrained, emotional, and external eating scores as compared to the males (*p* < 0.05). Cronbach’s alpha was 0.91 for restrained eating, 0.95 for emotional eating, and 0.79 for external eating.

**Table 2:** DEBQ scores of underweight and normal-weight subjects

	Male		Female	
	Underweight (*n* = 33)	Normal weight (*n* = 210)	Underweight (*n* = 61)	Normal weight (*n* = 226)
Restrained	1.8 ± 0.6^ab^	2.2 ± 0.8^c^	2.6 ± 0.9^a^	3.0 ± 0.8
Emotional	1.9 ± 0.9	1.8 ± 0.6^c^	2.2 ± 0.9	2.3 ± 1.0
External	3.0 ± 0.7^b^	3.1 ± 0.7^c^	3.4 ± 0.7	3.4 ± 0.7

Values are means ± standard deviations

^a^Significantly different from normal weight in each gender (Student’s *t* test with Bonferroni correction)

^b^Significantly different from female underweight (Student’s *t* test with Bonferroni correction)

^c^Significantly different from female normal weight (Student’s *t* test with Bonferroni correction)

**Fig. 1:**
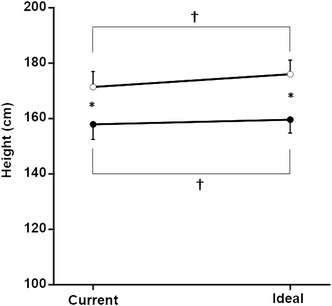
Comparison of current and ideal height. Male current height and ideal height are shown by *open circles*, and the female current height and ideal height are shown by *closed circles*. There was also a significant interaction effect between current-ideal and gender on height (*F* = 61.7, *p* < 0.001). There were also significant main effects of current-ideal and gender on height *single asterisk* significant difference (*p* < 0.05) between males and females. *Dagger* significant difference (*p* < 0.05) between current height and ideal height

**Fig. 2:**
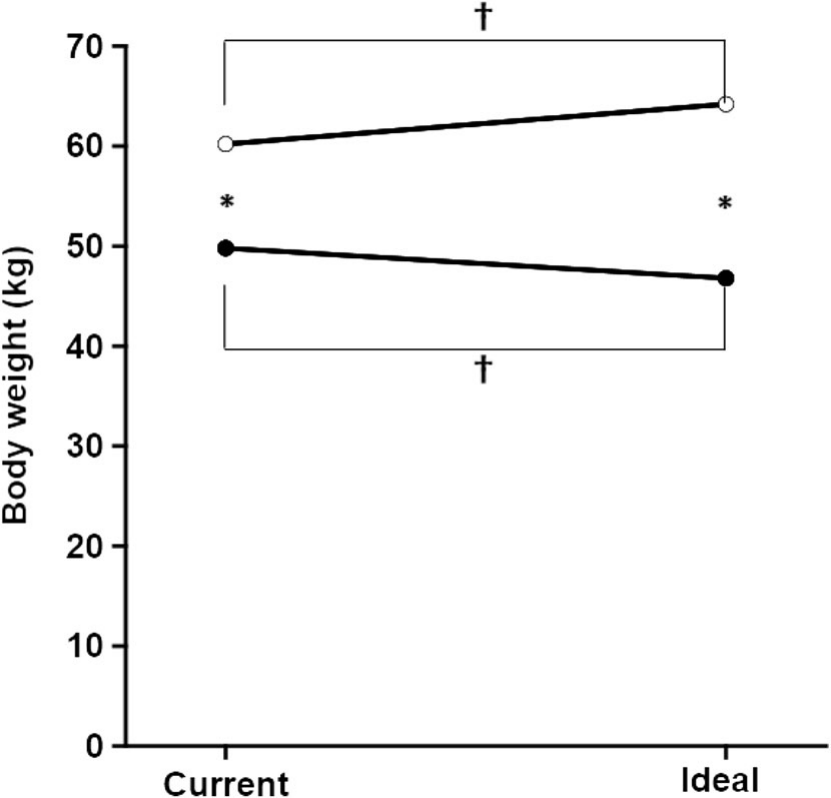
Comparison of current and ideal body weight. Male current body weight and ideal body weight are shown by *open circles*, and the female current body weight and ideal body weight are shown by *closed circles*. There was also a significant interaction effect between current-ideal and gender on body weight (*F* = 214.7, *p* < 0.001). There were also significant main effects of current-ideal and gender on body weight. *Single asterisk* significant difference (*p* < 0.05) between males and females. *Dagger* significant difference (*p* < 0.05) between current body weight and ideal body weight

**Fig. 3:**
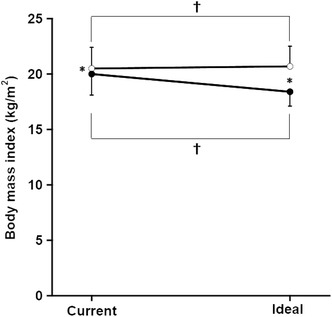
Comparison of current and ideal body mass index. Male current body mass index and ideal body mass index are shown by *open circles*, and the female current body mass index and ideal body mass index are shown by *closed circles*. There was also a significant interaction effect between current-ideal and gender on body mass index (*F* = 158.5, *p* < 0.001). There were also significant main effects of current-ideal and gender on body mass index. *Single asterisk* significant difference (*p* < 0.05) between males and females. *Dagger* significant difference (*p* < 0.05) between current body mass index and ideal body mass index

**Fig. 4:**
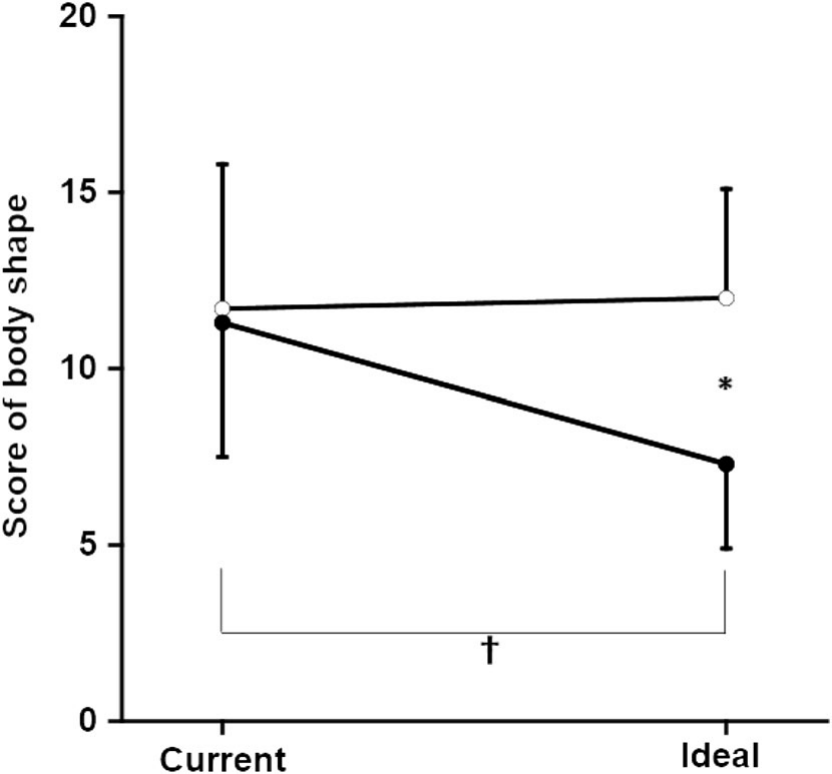
Comparison of current and ideal body shape. Male current body shape and ideal body shape are shown by *open circles*, and the female current body shape and ideal body shape are shown by *closed circles*. There was also a significant interaction effect between current-ideal and gender on body shape (*F* = 163.2, *p* < 0.001). There were also significant main effects of current-ideal and gender on body shape. *Single asterisk* significant difference (*p* < 0.05) between males and females. *Dagger* significant difference (*p* < 0.05) between current body shape and ideal body shape

Discrepancies in height, weight, BMI, and BSh between the participants’ current data and their ideal values are shown in Figs. 1, 2, 3, 4. The two-way repeated measures ANOVA showed that there was a significant interaction effect between gender and height discrepancy (*F* = 61.7, *p* < 0.001). Both gender and height discrepancy had a significant main effect on height. After a post hoc test, both height and ideal height in the males were significantly higher than in the females. The ideal heights of both the males and females were significantly higher than their current heights.

The two-way repeated measures ANOVA also revealed a significant interaction effect between gender and weight discrepancy (*F* = 214.7, *p* < 0.001). Both gender and weight discrepancy had a significant main effect on weight. After a post hoc test, weight and ideal weight in males were significantly larger than in the females. The ideal weight values were significantly larger than the current weight values in the males, whereas in the females, the ideal weight values were significantly smaller than the current weight values.

A significant interaction effect between gender and BMI discrepancy (*F* = 158.5, *p* < 0.001) was also observed. Both gender and BMI discrepancy had a significant main effect on BMI. After a post hoc test, the current BMI and ideal BMI were significantly larger in the males than in the females. In the males, the ideal BMI values were significantly larger than the BMI values, whereas in the females the ideal BMI values were significantly smaller than the BMI values.

There was also a significant interaction effect between gender and BSh discrepancy (*F* = 163.2, *p* < 0.001). Both gender and BSh discrepancy had a significant main effect on BSh. After a post hoc test, the ideal BSh values reported by the males were significantly larger than those reported by the females. In the females, the ideal BSh was significantly smaller than body shape.

BMI was significantly positively correlated with the score of restrained and external eating in males (restrained eating: *r* = 0.213, *p* = 0.001; external eating: *r* = 0.183, *p* = 0.004). In females, BMI was significantly positively correlated with the score of restrained and emotional eating (restrained eating: *r* = 0.217, *p* < 0.001; emotional eating: *r* = 0.148, *p* = 0.012).

Table 3 shows the results of our multiple linear regression analysis between the DEBQ scores and the discrepancies of weight, BMI and perception of BSh, adjusting for height. In the males, restrained eating was significantly negatively associated with the discrepancy of weight (*β* = − 0.185, *p* = 0.005), the discrepancy of BMI (*β* = − 0.363, *p* < 0.001), and the discrepancy of BSh (*β* = − 0.455, *p* < 0.001). External eating was negatively associated with the discrepancy of BMI (*β* = − 0.124, *p* = 0.049).

**Table 3:** DEBQ scores and discrepancy of physical index

	Discrepancy of weight		Discrepancy of BMI		Discrepancy of body shape	
	*β*	*p* value	*β*	*p* value	*β*	*p* value
Male						
Restrained	−0.185	0.005*	−0.363	<0.001*	−0.455	<0.001*
Emotional	0.049	0.470	0.092	0.155	0.071	0.255
External	−0.079	0.233	−0.124	0.049*	−0.034	0.579
Female						
Restrained	−0.159	0.007*	−0.236	<0.001*	−0.276	<0.001*
Emotional	−0.107	0.114	−0.145	0.023*	−0.157	0.015*
External	−0.025	0.709	0.034	0.602	−0.062	0.337

*β* standard coefficient in multiple linear regression analysis, adjusting for height

*BMI* body mass index

* Significantly correlated with a discrepancy of physical index

Among the females, restrained eating was negatively associated with the discrepancy of weight (*β* = − 0.159, *p* = 0.007), the discrepancy of BMI (*β* = − 0.236, *p* < 0.001), and the discrepancy of BSh (*β* = − 0.276, *p* < 0.001). Emotional eating was negatively associated with the discrepancy of BMI (*β* = − 0.145, *p* = 0.023) and the discrepancy of BSh (*β* = − 0.157, *p* = 0.015).

## Discussion

We attempted to clarify the relationship between eating behavior and body image in Japanese university students. Our main findings show that the ideal weight and ideal BMI were higher than the current weight and BMI in males, but lower in females. On the other hand, the females’ ideal body shape was smaller than their perception of current body shape. Restrained eating, emotional eating, and external eating in the DEBQ were higher in the females than the males among the normal-weight participants, and restrained eating and external eating were higher in the females than the males among the underweight participants. In addition, in the results of multiple linear regression analysis adjusting for height, higher score of the restrained eating showed less discrepancy of weight, BMI, and body shape in both the males and females. Emotional eating was negatively associated with the discrepancy in BMI and body shape only among the females.

### Gender differences in ideal body image

We asked the participants to state their ideal height in addition to their ideal body weight and ideal body shape. Few studies have included ideal height as a study point, and here we found that the questionnaire’s item about ideal height helped to clarify the gender difference in ideal body shape. Namely, the ideal height, ideal weight, ideal BMI, and ideal body shape were all larger than the current values in the males, whereas in the females, the ideal body weight and ideal body shape were lower than the current values, although ideal height was higher than the current height.

In previous reports, females tended to aspire to a slender body shape [25–27], which is consistent with our present findings. It is interesting that young Japanese females still tend to aspire to a lean body even though they are not obese as compared with females in Europe or the US [3, 27]. The reason why non-obese Japanese females aspire to a lean body and the reason why underlying the gender difference in ideal body shape are not yet clear. Females might prefer a lean body shape irrespective of their height or current body shape. In males, McCreary and Sasse [15] defined the drive for muscularity, which is a desire to enhance one’s musculature, and other studies with similar results have been published [16–18]. This may be one possible reason for the present results. Further investigations are needed to draw conclusions about the direction and gender differences in body shape.

### Eating behavior and anthropometric index and body image

Almost all of the previous studies that used the DEBQ reported that only restrained eating was used to examine the association of DEBQ with body image. In the present study, we examined not only restrained eating, but also all three eating indexes of the DEBQ to explore the gender difference. It has been reported that the scores of restrained eating were higher in females than in males [28–30], which are consistent with the present study. Nguyen-Rodriguez et al. [31] reported that scores of emotional eating were not different between boys and girls in junior high school, whereas the score of emotional eating in females was significantly higher than in males in the present study. This disparity indicates that age may have some relations with emotional eating. Indeed, previous studies in adults showed that the scores of emotional eating in females were significantly higher than those in males [29, 30].

Moreover, the scores on all three eating indexes were higher in the females than the males among the normal-weight participants, and restrained eating and external eating were higher in the females than the males among the underweight participants. In light of these results, it appears that both normal-weight and underweight females may be more sensitive to eating than their male counterparts.

Our multiple linear regression analysis showed that in the females, restrained eating was negatively associated with the discrepancies in weight, BMI, and body shape. Emotional eating in females was also negatively associated with the discrepancies of BMI and body shape. These results indicate that restrained and emotional eating were associated with a drive for thinness in females, which is consistent with previous reports [10, 32, 33]. Among the present study’s male participants, restrained eating was negatively associated with the discrepancies in weight, BMI, and body shape, similar to the present results for the females. On the other hand, emotional eating was not significantly associated with any discrepancies in the males. External eating was significantly associated with the discrepancy of BMI.

The reason for the gender differences in the present results is unclear. Emotional eating is most often defined as eating in response to negative affect [34]. Nguyen-Rodriguez et al. showed that emotional eating was positively associated with negative mood, such as perceived stress and worries, only in females [31]. Thus, females may be more sensitive to emotional eating than males. This concept should be studied more precisely in the future.

### Limitations

The limitations of this study should be noted. First, the samples were collected from a limited area. Second, the present participants were all Japanese students. Regarding the cut-off points of underweight and overweight, WHO criteria are also used for Japanese people. However, in general, Japanese individuals are considerably thinner and shorter than age-matched North American and European individuals. Therefore, the number of overweight subjects was not enough large to analyze, and it may be difficult to generalize the present results.

### Conclusions

In the present study, the ideal weight and ideal BMI values were higher than the current weight and current BMI in the males, but lower in the females, whereas ideal body shape was smaller than the perception of current body shape in the females, but not significantly different in the males. In addition, restrained eating, emotional eating, and external eating as measured by the DEBQ were higher in the females than in the males. Among the normal-weight participants, all three of these eating indexes were higher in the females as compared to the males, and among the underweight participants, restrained eating and external eating were higher in the females than the males. Taken altogether, our results indicate that at least in Japanese university students, the gender differences regarding ideal body shape are related to eating behavior.
